# The Convention of Medical Colleges

**Published:** 1876-08

**Authors:** 


### Editorial.

---

The Convention of Medical Colleges.—Delegates from twenty-three medical colleges met in Philadelphia on the second day of June, and as was seen from the proceedings published in the July number of this journal, held their first meeting. In addition to the twenty-three colleges represented by delegates, there were ten or fifteen represented by letters, in which they took occasion to express their entire sympathy with the movement. As a majority of the medical schools were not represented by delegates upon the floor, much of the business brought before the convention was deferred until its next meeting; and for the same reason the action taken upon many of the questions was not so decided as would have been, and as doubtless will be when the convention is permanently organized, and is the accepted law-making body that is in the future to give direction to medical teaching in the United States. Enough was accomplished, however, to demonstrate the practicability of the movement, and above all to impress most forcibly those present with the absolute necessity of some such organization to control medical teaching and put a stop to the strife and disgraceful contests which have so frequently occurred within the past few years.

Since the American Medical Association declined to receive delegates from medical colleges, and to extend any supervision, either moral or otherwise, over the schools, they have been left without any law-making power; each school being a law unto itself, and are in the same condition that the medical profession would be without a code of ethics and a body with power to decide all ethical questions. The result has been anything but encouraging.

In some localities in the South and West, if reports be true, the whole system of teaching has degenerated to the extent that it would appear that the prominent feature is a scramble for numbers. If the half of what schools in certain localities publicly say of each other be true, the great object is not so much the preparation of the student for the responsible duties of the profession, or to guard its portals, as to increase the size of the classes in attendance and diminish those of rival institutions. While we regard an ambition to build up a large and flourishing school as laudable and altogether legitimate and proper when all interests are protected; and while much that is said by rival schools is not strictly founded on fact, or at most greatly exaggerated, still it is evident to all thinking men not directly engaged in such contests that a point has been reached that calls for action—for reform.

And what shall this action be? The plan of leaving each school to make its own laws, and to conduct its affairs in its own way, without reference to other colleges or the good of the profession, has been sufficiently tested to make prominent its startling defects. Were we all perfect and of one opinion, there would be no differences, and all would move on without a jar, and nothing in the form of a code or law would be required; but unfortunately this is not the case, and since the world began man has required restraints.

A code to govern those engaged in medical teaching is just as essential as to those engaged in the practice of medicine, if we hope to preserve the dignity of medical teaching. It is no less undignified to underbid in medical teaching than in the practice of medicine, and no less dishonorable to increase a class to the detriment of a rival school by undue and unfair means than for a practitioner to increase his business by like unfair and dishonorable practices. To preserve then the dignity of teaching, a code of ethics to govern medical colleges, with a body with law-making power to decide ethical questions, is just as essential as the code of ethics governing the profession in preserving the dignity of the practice of medicine.

While this position, by some, may be regarded as extreme, still we feel that it is warranted and more than demonstrated by what has actually transpired within the past few years. With such convictions we hailed with pleasure the call for a Convention of Medical Colleges, which was proposed, not for the purpose of pulling down or injuring some schools to build up others, as some who were not in attendance have been foolish enough to suggest, but called in the interest of the dignity and honor of medical teaching and the medical profession. How any one engaged in teaching, and who has the good of the profession at heart, can oppose this movement is more than we can solve.

Let every school send one or more delegates to the next convention, and let them go prepared to make concessions and to adopt in good faith whatever the majority in council, after mature deliberation, may decide is best for all, and our word for it there will be an end of the strife and disgraceful contests which have so frequently disturbed and disgusted the profession for the past few years.

